# Synergistic inhibition of autophagic flux and induction of apoptosis in cervical cancer cells by Mito-TEMPO and hyperthermia

**DOI:** 10.1265/ehpm.25-00204

**Published:** 2025-09-05

**Authors:** Yu-Mei Li, Qing-Li Zhao, Ryohei Ogawa, Tatsuji Mizukami, Yu Song, Zheng-Guo Cui, Jun-Ichi Saitoh, Kyo Noguchi

**Affiliations:** 1 Division of Radiation Oncology, Department of Radiology, Faculty of Medicine, University of Toyama, Toyama 930-0194, Japan; 2 Department of Environmental Health, University of Fukui School of Medical Sciences, Fukui 910-1193, Japan

**Keywords:** Hyperthermia, Mito-TEMPO, Apoptosis, Autophagy, Endoplasmic reticulum stress

## Abstract

**Background:**

Hyperthermia (HT), while a cancer treatment approach, isn’t always effective alone. Therefore, identifying hyperthermia enhancers is crucial. We demonstrated that Mito-TEMPO ([2-[(1-Hydroxy-2,2,6,6-tetramethylpiperidin-4-yl) amino]-2-oxoethyl]-triphenylphosphanium, MT) acts as a potent thermosensitizer, promoting cell death in human cervical cancer (HeLa) cells.

**Methods:**

Cells were pretreated with 0.4 mM MT for 5 minutes, followed by exposure to hyperthermia (42 °C for 60 minutes). The impacts of MT/HT on cell viability, proliferation, apoptosis, endoplasmic reticulum (ER) stress, apoptosis-related proteins and autophagy, autophagy-related proteins expression were measured. The relationships between autophagy and apoptosis were further investigated using the specific autophagy inhibitor chloroquine (CQ) and the autophagy inducer rapamycin (Rapa).

**Results:**

The combined treatment reduced the mitochondrial membrane potential (MMP) and increased ROS production. It also upregulated the pro-apoptotic protein Bax and downregulated anti-apoptotic proteins such as Bcl-2 and MCL-1. As a result, Caspase-3 was activated. Additionally, the combined treatment upregulated the expression of p-PERK/PERK, ATF-4, CHOP proteins. Moreover, the combined treatment also increased the expression of LC3 II and p62, decreased expression of LAMP 1 and Cathepsin D and increased lysosomal pH, indicating coordinated changes in autophagy regulation. Notably, intensification of apoptosis induced by the combined treatment was observed with CQ, whereas attenuation was seen with Rapa.

**Conclusions:**

MT effectively enhanced HT-induced apoptosis in HeLa cells. Elevated ER stress and interruption of autophagy flux are the possible underlying molecular mechanisms for this phenomenon. These findings suggested MT can act as a potential thermosensitizer, highlighting its versatility in cancer treatment strategies.

**Supplementary information:**

The online version contains supplementary material available at https://doi.org/10.1265/ehpm.25-00204.

## Introduction

Apoptosis is a tightly regulated process of programmed cell death that plays a pivotal role in development, tissue homeostasis, and the removal of damaged or abnormal cells. In cancer therapy, apoptosis is crucial for targeting and destroying cancer cells. By inducing the self-destruction of cancer cells, enhancing immune responses, and overcoming drug resistance, apoptosis serves as a crucial mechanism for effectively inhibiting tumor growth and metastasis [1]. During cancer therapy, the disruption of intracellular homeostasis in cancer cells induces multiple stress responses that are crucial to the therapeutic outcome. Key disruptions include metabolic dysregulation, oxidative stress, and ER stress. Metabolic pathways, particularly mitochondrial function, are often impaired, leading to reduced ATP levels and the activation of cell death pathways such as apoptosis and autophagy. Mitochondria play a central role in regulating apoptosis by acting as the key site where signals from pro-apoptotic proteins (e.g., Bax and Bak) and anti-apoptotic proteins (e.g., Bcl-2 and MCL-1) converge to determine cell fate [2]. Oxidative stress, marked by a sharp increase in reactive oxygen species (ROS), leads to damage of DNA, proteins, and lipids, thereby promoting apoptosis.

Cancer treatments frequently cause the accumulation of misfolded proteins in the endoplasmic reticulum (ER), activating the unfolded protein response (UPR). While UPR aims to restore protein homeostasis, prolonged ER stress can trigger apoptosis via pathways such as CHOP activation [3]. These stress responses typically lead to cancer cell death; however, some cells can adapt by enhancing protective mechanisms, such as antioxidant defenses, upregulating the UPR and activating autophagy, which may contribute to therapeutic resistance. Autophagy is a process in which cells maintain intracellular homeostasis by degrading and recycling their own organelles and proteins in response to conditions such as starvation or stress. Autophagy plays a complex role in regulating the balance between cell survival and death, as it can either protect cells from damage or, in certain circumstances, promote cell death [4, 5]. Under ER stress, autophagy inhibition can exacerbate cell death by promoting the accumulation of misfolded proteins and damaged organelles within the ER. Typically, when autophagy is suppressed, cells are unable to effectively manage the excessive protein load and ER dysfunction associated with prolonged stress. This failure to alleviate ER stress results in enhanced cellular damage, ultimately leading to increased apoptosis or necrosis [6].

Hyperthermia (HT) involves applying specific temperature ranges to localized tissues through various methods for a defined duration. Extensive experimental and clinical studies have demonstrated the efficacy of hyperthermia in efficiently treating tumors [7–9]. This process externally elevates the local temperature from 41 °C to 45 °C [10]. Hyperthermia can directly induce tumor cell necrosis or activate specific pathways leading to programmed cell death, such as apoptosis, pyroptosis, necroptosis, and ferroptosis, depending on the temperature [11–14]. Studies indicate that simulated tumor hyperthermia at 42 °C, in comparison to the control group at 37 °C, significantly inhibits the growth of colorectal cancer (CRC) cells [15]. Challenges in hyperthermia application arise when heating large areas or treating tumors located deep within the body, leading to uneven heating and insufficient temperature levels for effective tumor cell eradication [16]. Thus, the effectiveness of hyperthermia alone in kill tumor cells may be limited, increasing the burden on patients. Therefore, the significance of hyperthermia lies in its ability to produce synergistic effects when combined with radiotherapy and chemotherapy [14, 17–20], enhancing patient sensitivity to these treatment modalities.

**Fig. 1:**
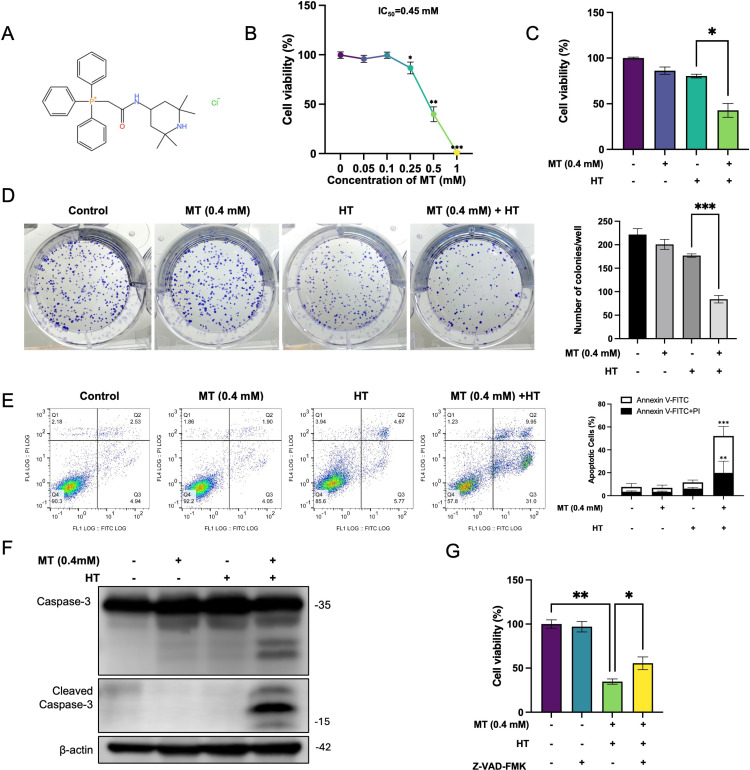
MT enhances HT-Induced apoptosis in HeLa cells and suppresses proliferation. (A) The structural formula of MT. (B) HeLa cells were continuously exposed to various concentrations of MT for 24 hours. Cell viability analysis was performed using Cell Counting Kit-8 assay. (C) Cells were pre-treated with or without MT (0.4 mM) for 5 minutes and then exposed to HT (42 °C for 60 minutes) or not, then MT washed out after HT treatment and incubated in fresh medium at 37 °C. Cell viability analysis was performed using Cell Counting Kit-8 assay after 24 hours. (D) Antiproliferative effect of this combination analysis was performed using Giemsa stain assay after 10 days. (E) flowcytometry using Annexin V-FITC/PI Apoptosis Detection Kit after 24 hours. (F) Caspase-3 and Cleaved Caspase-3 expression was determined using western blotting. (G) Cells were pre-treated with 100 µM Z-VAD-FMK, a pan-caspase inhibitor, for 1 hour and exposed to HT (42 °C for 60 minutes) in the presence of MT (0.4 mM), Cell viability analysis was performed using Cell Counting Kit-8 assay after 24 hours. The data in each bar graph are presented as the means standard errors of the means (SEM). *P < 0.05, **P < 0.01, ***P < 0.001.

Thermal sensitizers represent a class of pharmaceutical agents capable of significantly enhancing the efficacy of hyperthermic treatments [21]. Numerous researchers have explored sensitizers with low toxicity that efficiently amplify the thermal lethality towards cancer cells [22–26]. Tempo as an antioxidant, capable of effectively scavenging reactive oxygen species (ROS) and protecting cells from oxidative stress-induced damage. Our previous studies have shown that Tempo, when combined with hyperthermia (HT), can enhance HT-induce cell death. Preliminary investigations revealed that Tempo combined with 44 °C heat treatment promotes apoptosis in U937 cells [25, 27]. Mito-TEMPO (MT) (Fig. 1A) is a hybrid substance combining the antioxidant piperidine nitroxide Tempo with the lipophilic cation triphenylphosphonium. This unique composition allows MT to easily cross lipid bilayers and accumulate in mitochondria by several hundred folds, where it effectively scavenges mitochondrial superoxide (O^2−^) [28, 29]. This unique property makes MT a valuable tool in the investigation of mitochondrial oxidative stress. Previous studies have demonstrated the significant protective effects of MT in various experimental models. For instance, in models of neurodegenerative diseases, MT shown to enhances neuronal resilience against glutamate cytotoxicity by directly scavenging free radicals and modulating excessive autophagy signaling pathways [30]. In cardiovascular disease models, MT mitigates myocardial cell damage and preserves cardiac function [31, 32]. Additionally, MT has shown potential in cancer research by inhibiting cancer cell growth and metastasis through the reduction of oxidative stress-induced cellular damage, A study by Shetty et al. showed that MT provided significant protection against NDEA-induced hepatocarcinogenesis, possibly through inhibition of Mitochondrial ROS (mtROS), although further research is necessary to elucidate its mechanisms across different stages of cancer development [28].

To date, there have been no reports on MT as a sensitizer in hyperthermia. In this study, it is the first time to report that the combination of MT with HT (42 °C for 60 minutes) promotes cell death in HeLa cells. Herein, we report that MT, acting as a thermal sensitizer, influences lysosomal function, inhibits autophagic flux, concurrently inhibit mitochondrial function, and facilitates the apoptosis induced by hyperthermic treatment. Those findings provide new insights into the mechanisms by which MT enhances hyperthermia and offer scientific evidence for the development of more effective cancer therapies in the future.

## Material and methods

### Regents and antibodies

MT was purchased from Sigma Aldrich (Burlington, MA, USA). Primary antibodies against Caspase-3 (9662S), Cleaved Caspase-3 (9661S), Beclin-1 (34957), p62 (5114S), ATG5 (12994S), Sirt-3 (5490S), SOD-2 (13194S), PERK (3192S), p-PERK (3179S), ATF-4 (11815S), CHOP (5554S), p-JNK (9255S), JNK (9252S), p-p38 (9211S), p38 (9212S), Bax (2772), Bid (2002), MCL-1 (39334S), Bcl-2 (2870), Cathepsin D (2284S), and secondary anti-mouse (7076S) and anti-rabbit (7074S) antibodies were purchased from Cell Signaling Technology (Danvers, MA, USA). Anti-IRE1 (ab54692) were purchased from Abcam (Cambridge, UK). Anti-HSC-70 (SC-7298), LAMP 1 (SC-20011) antibodies were purchased from Santa Cruz Biotechnology (Santa Cruz, CA). Anti-LC3 (PD014), HSP-70 (SR-B810) were purchased from MBL (Medical & Biological Laboratories, JP).

### Cell culture and treatment

The human cervical cancer (HeLa) cells were purchased from the Human Sciences Research Resource Bank (Japan Human Sciences Foundation) and were grown in D-MEM medium (Wako Pure Chemical Corporation) supplemented with 10% heat-inactivated fetal bovine serum (SIGMA Life Science, Burlington, MA, USA) at 37 °C in humidified air with 5% CO_2_. For stimulation, HeLa cells were seeded onto plate at an indicated density of cells and incubated overnight, the cells were then pre-treated with or without MT (0.4 mM) for 5 minutes, followed by co-treatment with hyperthermia (42 °C) using a water bath for 60 minutes. After HT treatment, MT was washed out, and fresh complete medium was added before incubation at 37 °C.

### Cell viability assay

HeLa cells were seeded onto 96-well, flatbottomed plate at a density of 6 × 10^3^ cells/well with 90 µL complete medium and incubated at 37 °C overnight. After the desired treatment, cells were incubated for an additional 2 hours with 10 µL Cell Counting Kit-8 (CCK-8; Dojindo Laboratories, Inc. Kumamoto, Japan) solution. The absorbance at 450 nm was measured using an iMark™ Microplate Absorbance Reader (Bio-Rad Laboratories, Inc. Hercules, CA, USA).

### Cell colony formation

Cells (5 × 10^2^ per well) were seeded in 6-well plates and cultivated in indicated media for 10 days after the desired treatment. Subsequently, the media was removed, cells were washed twice in PBS and incubated with methanol and acetic acid (3:1) for 30 minutes at 4 °C, after drying, staining was performed using 5% Giemsa solution (pH 6.8) for 30 minutes at room temperature [33]. Plates were thoroughly washed with water and air-dried at room temperature.

### Apoptosis detection with Annexin V-FITC/PI stain

Cells (2 × 10^5^ per well) were seeded in 12-well plates and were cultivated in indicated media for 24 hours after the desired treatment. Subsequently, cells were harvested and washed twice with PBS and then cells were stained using Annexin V/PI apoptosis detection kit (Immunotech, Marseille, France) for 20 minutes at room temperature according to manufacturer’s instructions, the fluorescence emission of all probes was analyzed using flow cytometry. A total of 1 × 10^4^ events were analyzed for each sample using an Epics XL Flow Cytometer, Beckman-Coulter, Inc. [22].

### Western blotting analysis

After the desired treatment, cells were washed three times with PBS, and the total protein was extracted using RIPA lysis buffer containing protease and phosphatase inhibitors. The cell extracts were centrifuged at 12,000 × g at 4 °C for 10 minutes. The supernatants were transferred to 1.5 mL Eppendorf tubes. The concentrations of the proteins were measured using the Bradford Protein Assay Kit at 595 nm on a microplate reader. Twenty micrograms of protein per sample were electrophoresed at 20 mA for 90 minutes, followed by transfer to PVDF membranes (MilliporeSigma, Burlington, MA, USA) at 150 mA for 90 minutes. Next, the membranes were blocked with Blocking One (Nacalai Tesque, Kyoto, Japan) for 1 hour, and the specific primary antibody was incubated 2 hours at room temperature. After washed three times with TBST, the secondary antibody was added for 1 hour at room temperature. Finally, the target proteins were detected using an enhanced chemiluminescent (ImmunoStar LD) kit (Fujifilm Wako Pure Chemical Corporation, Japan) in accordance with the manufacturer’s instructions. The band densities were measured using Image Studio Lite (LI-COR Biosciences, Lincoln, NE, USA) [27].

### Immunofluorescence

Cells (2 × 10^4^ per well) were seeded in 8-well glass chamber slide and were cultivated in indicated media for 24 hours after the desired treatment. Subsequently, the media was removed, cells were washed twice in PBS, then cells were fixed with 4% paraformaldehyde in PBS for 20 minutes, permeabilized with 0.1% Triton X-100 in PBS for 15 minutes, blocked with Blocking buffer (Blocking One, Nacalai Tesque, Japan) for 1 hour, and incubated with primary antibody overnight at 4 °C. After incubation, the cells were flooded with secondary antibody for 1 hour, then stained with DAPI (6-diamidino-2-phenyl-indole dihydrochloride). Fluorescent images were observed using a microscope (Olympus, Center Valley, PA, USA, Center Valley, PA, USA) equipped with appropriate filters.

### Detection of ROS levels

Cells (2 × 10^5^ per well) were seeded in 12-well plates and were cultivated in indicated media for 6 hours after the desired treatment. Subsequently, cells were harvest and washed twice in PBS and then cells were incubated with 2′,7′-dichlorodihydrofluorescein diacetate (DCFH-DA) (Molecular Probes, Eugene, OR, USA) and dihydroethidium (DHE) (Molecular Probes, Eugene, OR, USA) staining respectively for 30 minutes at 37 °C, the fluorescence emission of all probes was analyzed using flow cytometry. A total of 1 × 10^4^ events was analyzed for each sample using an Epics XL Flow Cytometer, Beckman-Coulter, Inc.

Cells (2 × 10^4^ per well) were seeded in 8-well glass chamber slide and were cultivated in indicated media for 6 hours after the desired treatment. Subsequently, the media was removed, cells were washed twice in PBS, and then cells were incubated with DCF and DHE staining respectively for 30 minutes at 37 °C. Fluorescent images were observed using a laser-scanning confocal microscope (Olympus, Center Valley, PA, USA) equipped with appropriate filters [18].

### Mitochondrial membrane potential

Cells (1 × 10^6^ per well) were seeded in 6-well plates and cultivated in indicated media for 24 hours after the desired treatment. Subsequently, cells were harvest and washed twice in PBS and then cells were incubated with 10 nM tetramethylrhodamine methyl ester (TMRM) (Molecular Probes, Eugene, OR) for 15 minutes at 37 °C in PBS, the fluorescence emission of all probes was analyzed using flow cytometry. A total of 1 × 10^4^ events was analyzed for each sample using an Epics XL Flow Cytometer, Beckman-Coulter, Inc.

Cells (2 × 10^4^ per well) were seeded in 8-well glass chamber slide and were cultivated in indicated media for 24 hours after the desired treatment. Subsequently, the media was removed, cells were washed twice in PBS, and then cells were incubated with TMRM and 1 µM MitoTracker green (Thermo Fisher Scientific) staining respectively for 30 minutes at 37 °C. Fluorescent images were observed using a laser-scanning confocal microscope (Olympus, Center Valley, PA, USA) equipped with appropriate filters.

### Examination of GFP-RFP-LC3B punctation

Cells (2 × 10^4^ per well) were seeded in 8-well glass chamber slide and transiently transfected with the Premo™ Autophagy Tandem Sensor RFP-GFP-LC3B adenoviruses as per manufacturer’s protocols. After 24 hours, cells were treated with designated treatments, followed by fixation using 4% formaldehyde for 15 minutes at room temperature. Visualization of LC3B puncta was performed using a fluorescent microscope (Olympus, Center Valley, PA, USA) and analyzed using FIJI software.

### Lysosomal tracking

Cells (2 × 10^4^ per well) were seeded in 8-well glass chamber slide and cultivated in indicated media for 24 hours after the desired treatments. Following this, they were incubated with LysoTracker Red DND-99 (50 nM) (Thermo Fisher Scientific) for 30 minutes at 37 °C to visualize lysosomes. Nuclei counterstaining was subsequently conducted with Hoechst 33342 (Thermo Fisher Scientific). Fluorescent images were observed using a laser-scanning confocal microscope (Olympus, Center Valley, PA, USA) equipped with appropriate filters.

### Statistical analysis

GraphPad Prism 8.0 was employed to visualize graphs and conduct data analyses, and the data were presented as the mean ± standard deviation (mean ± SD). The Student’s t-test was utilized to detect significant differences between groups, while the one-way or two-way analysis of variance (ANOVA) was applied for comparisons of multiple groups [33]. Statistical significance was determined at a level of P < 0.05, and significant differences were expressed as *P < 0.05, **P < 0.01 or ***P < 0.001.

## Results

### Combined treatment of MT with HT suppresses proliferation and enhanced apoptosis in HeLa cells

To investigate the cytotoxic effects of MT on HeLa cells, HeLa cells were continuously exposed to various concentrations of MT (0–1 mM) for 24 hours, then the cell viability assessed using CCK-8 assays. The results indicated that the growth inhibitory effect of MT on HeLa cells was dose-dependent manner, with an IC_50_ values of approximately 0.45 mM (Fig. 1B and Supplementary Table S1). To investigate the cytotoxic effects of MT combined with HT on cell proliferation and cell death, HeLa cells were treated with or without MT (0.4 mM) and/or HT (42 °C) for 1 hour, after the treatment, MT was washed out, and cells were incubated in fresh medium for additional 24 hours, then the cell viability assessed using CCK-8 assays. The results showed that the combination treatment significantly decreased cell viability compared to HT alone, as determined by the CCK-8 assay (Fig. 1C and Supplementary Table S2). Furthermore, the combination treatment significantly inhibited colony formation compared to HT alone (Fig. 1D). Next, we employed Annexin V-FITC/PI double staining. The results demonstrated that the induced cell death was due to apoptosis, as evidenced by flow cytometry (Fig. 1E) and Cleaved Caspase-3 (Fig. 1F). Additionally, the pan-caspase inhibitor Z-VAD-FMK counteracted the apoptosis induced by the combined treatment in HeLa cells (Fig. 1G). These results indicated that MT significantly enhanced HT-induce apoptosis in HeLa cells and suppresses proliferation in HeLa cells.

### MT combined with HT induces the loss of MMP and disrupts mitochondrial homeostasis

**Fig. 2:**
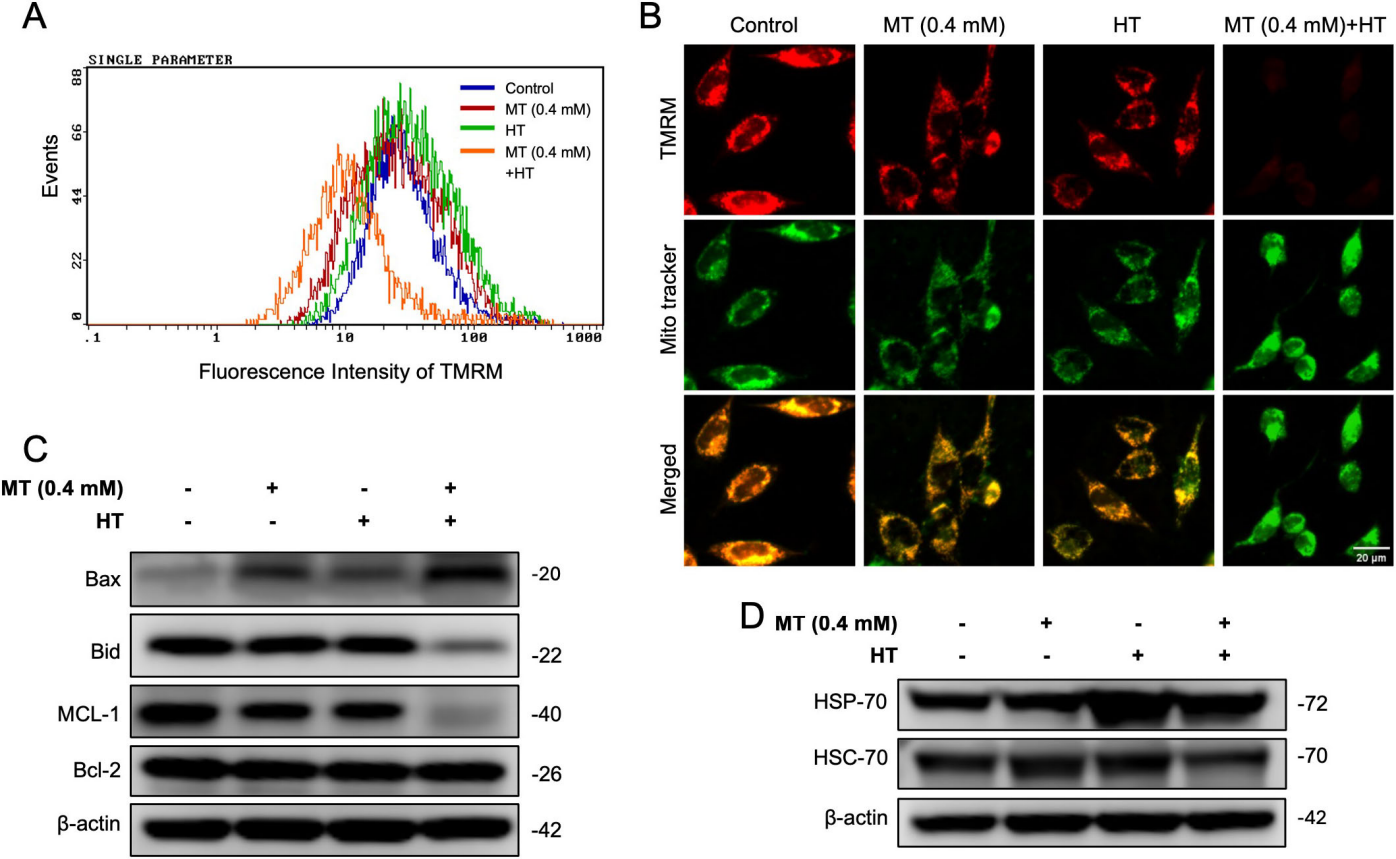
MT plus HT induces mitochondrial membrane potential loss in HeLa cells. Cells were pre-treated with or without MT (0.4 mM) treatment for 5 minutes, followed by exposed to HT (42 °C for 60 minutes). After HT treatment, MT was washed out, and fresh complete medium was added before incubation at 37 °C. After 24 hours incubation, changes in MMP levels were measured using TMRM fluorescence staining (MitoTracker, green; TMRM, red), analyzed using (A) flow cytometry and (B) fluorescence microscope. (C) The expression levels of Bcl-2 family proteins (Bax, Bid, MCL-1, Bcl-2) and (D) Heat shock proteins HSP-70, HSC-70 in the cells were detected by Western blotting.

Mitochondrial membrane potential (ΔΨm) is a universal selective indicator of mitochondrial function [34]. To elucidate the mechanism of MT combined with HT-induced apoptosis, we explored the effect of the combination on MMP in HeLa cells using TMRM fluorescent probe. Results showed that the MMP in cells treated with combination treatment was decreased significantly compared with HT treated alone (Fig. 2B). This result was also confirmed by flow cytometry (Fig. 2A). These results suggest that MT combined with HT induced a significant loss of MMP in HeLa cells.

Bcl-2 family proteins are essential players in the regulation of mitochondrial outer membrane permeabilization (MOMP), a pivotal event in apoptosis [35]. We also investigated protein expression levels of the members of Bcl-2 family protein. The results showed that combination treatment significantly increased the expression of Bax and decreased the expression of full length of Bid. Moreover, the expression levels of the MCL-1 were reduced in combination group. However, no substantial change was observed in the expression level of the protein Bcl-2 (Fig. 2C). Taken together, these results indicate that MT combined with HT could potentially alter the equilibrium between pro-apoptotic and anti-apoptotic expressions of Bcl-2 family proteins, as well as influence the depolarization of MMP.

Heat shock proteins (HSPs) are protective proteins produced by cells in response to stress, such as heat, oxidative stress, and infections [36]. HSPs are classified into different families based on their molecular weight, such as HSP-27, HSP-60, HSP-70, and HSP-90. While they are highly expressed during stress, some HSPs are also present under normal physiological conditions, such as HSC-70, playing vital roles in maintaining cellular homeostasis and protecting cells from damage [37]. We found that the expression levels of both HSP-70 and HSC-70 proteins were reduced in the combined treatment group compared to those in the heat treatment alone group (Fig. 2D).

### MT combined with HT increased generation of intracellular ROS

**Fig. 3:**
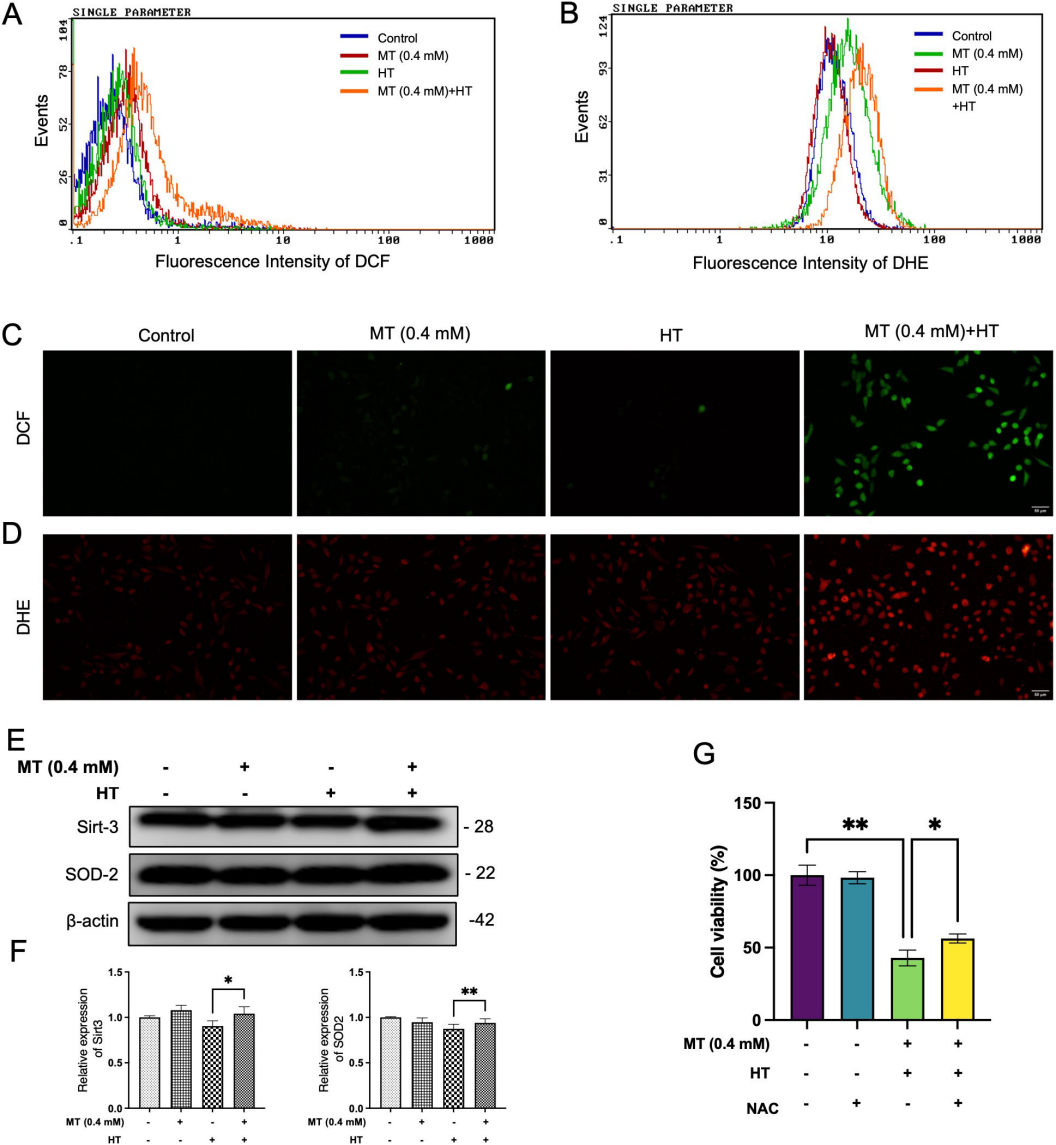
MT synergizes with HT to enhance ROS production in HeLa cells. Cells were pre-treated with or without MT (0.4 mM) treatment for 5 minutes, followed by exposure to HT (42 °C for 60 minutes). After HT treatment, MT was washed out, and fresh complete medium was added, with cells incubated at 37 °C. After 6 hours of incubation, cells were loaded with DCFH-DA for 30 minutes and analyzed using (A) flow cytometry and (C) fluorescence microscope. Cells were loaded with DHE for 30 minutes and analyzed using (B) flow cytometry and (D) fluorescence microscope. (E) The expression levels of ROS-related proteins Sirt-3 and SOD-2 in the cells were detected by Western blotting after 6 hours. (F) Quantification of immunoblotting data was performed using the FIJI program, normalized to β-actin as a loading control. (G) Cells were pre-treated with 1 mM NAC, a ROS inhibitor, for 1 hour and exposed to HT (42 °C for 60 minutes) in the presence of MT (0.4 mM), Cell viability analysis was performed using Cell Counting Kit-8 assay after 24 hours. The data in each bar graph are presented as the means standard errors of the means (SEM). *P < 0.05, **P < 0.01.

ROS are by-products of normal cellular metabolism. ROS play a dual role in cancer: they promote genetic alterations that contribute to tumor development and chemotherapy resistance, while elevated ROS levels induce cytotoxic effects that activate apoptosis [28]. We used 2′,7′-dichlorodihydrofluorescein diacetate (DCF-DA) and dihydroethidium (DHE) staining to measure intracellular ROS generation by flow cytometry and immunofluorescence. The results showed a significant increase in the fraction of cells with excessive generation of peroxide and superoxide following the combination treatment (Fig. 3A–3D). Additionally, no downregulation trend was observed in the expression of Sirt-3 and SOD-2 proteins in HeLa cells post 6-hour treatment (Fig. 3E–3F). Moreover, an antioxidant, NAC (N-acetyl-L-cysteine) could improve the viability of HeLa cells caused by combined treatment (Fig. 3G). These results indicated that the combination of MT and HT could increase oxidative stress, contributing the enhancement of the apoptosis in HeLa cells.

### MT combined with HT induces ER stress by MAPK pathway

**Fig. 4:**
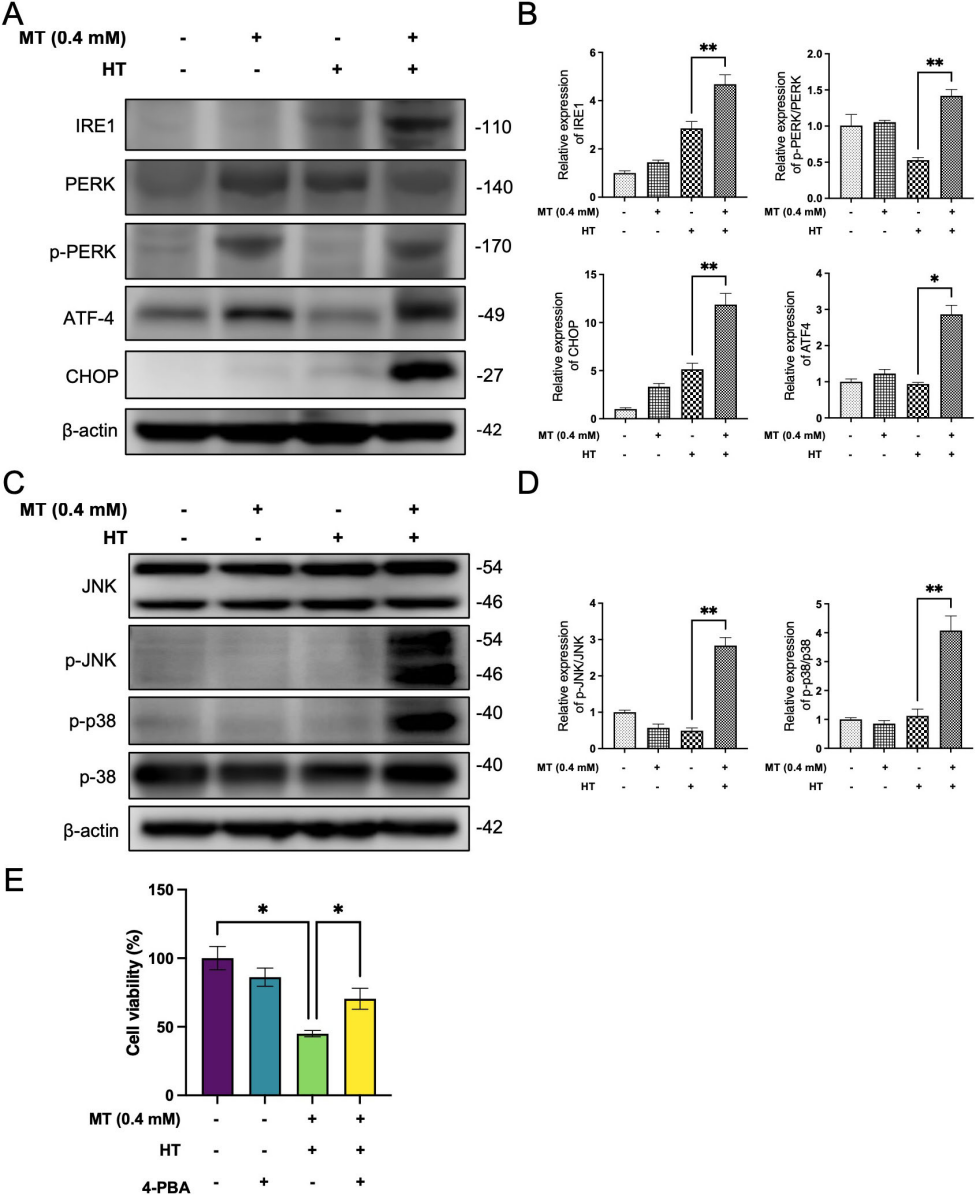
MT enhances HT-induced ER stress in HeLa cells. Cells were pre-treated with or without MT (0.4 mM) for 5 minutes, followed by exposure to HT (42 °C for 60 minutes). After HT treatment, MT was washed out, and fresh complete medium was added before incubation at 37 °C. (A) After 3 hours incubation, IRE1, PERK, p-PERK, CHOP and ATF-4 expression was determined using western blotting. (B) Quantification of immunoblotting data was performed using the FIJI program, normalized to β-actin as a loading control. After 1 hour and 6 hours incubation, (C) p-JNK, JNK and p38, p-p38 expression was respectively determined using western blotting. (D) Quantification of immunoblotting data was performed using the FIJI program, normalized to β-actin as a loading control. (E) Cells were pre-treated with 5 mM 4-PBA, an ER stress inhibitor, for 1 hour and exposed to HT (42 °C for 60 minutes) in the presence of MT (0.4 mM), Cell viability analysis was performed using Cell Counting Kit-8 assay after 24 hours. The data in each bar graph are presented as the means standard errors of the means (SEM). *P < 0.05, **P < 0.01.

Various pathological stresses, including oxidative stress, can induce ER stress. Although ER stress initially serves as a compensatory mechanism, prolonged ER stress can trigger cell death pathways in compromised cells [38, 39]. Therefore, we investigated the potential effect of MT combined with HT on ER activation. Combination treatment significantly upregulated markers of ER stress, including IRE1, p-PERK/PERK, ATF4 and C/EBP homologous protein (CHOP) (Fig. 4A–4B), suggesting that combination treatment induces ER stress through the PERK-ATF4-CHOP pathway in HeLa cells.

MAPKs are important downstream factors of ER stress [40]. Next, we investigated the possible role of MAPK pathways in the MT enhanced apoptosis. Phospho-JNK (p-JNK), JNK, phospho-p38 (p-p38), p38 levels were examined using western blot analysis. The results show that combination treatment significantly increased the expression of p-JNK/JNK and p-p38/p38 proteins (Fig. 4C–4D). Moreover, the ER stress inhibitor 4-Phenylbutyric acid (4-PBA) could improve the viability of HeLa cells caused by combined treatment (Fig. 4E). Taken together, these data demonstrated that ER stress is associated with the synergistic enhancement of apoptosis induced by MT and HT in HeLa cells.

### MT combined with HT impairs autophagic flux and lysosomes functionality in HeLa cells

**Fig. 5:**
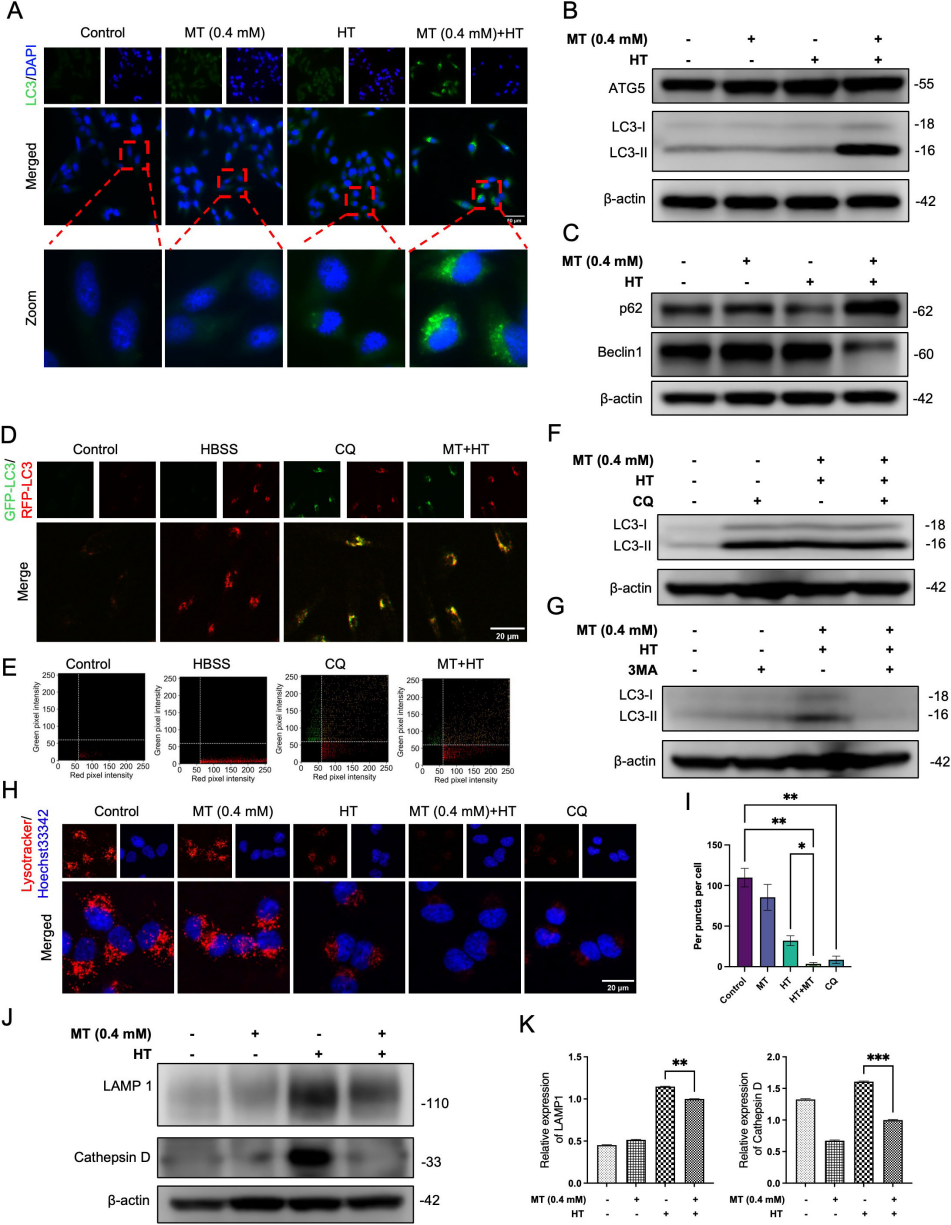
MT combined with HT impairs autophagic flux and lysosomes functionality in HeLa cells. LC3 protein was determined using (A) Immunofluorescence stain (LC3, green; DAPI, blue) and (B) western blotting. (C) p62 and Beclin1 proteins was determined using western blotting. Cells were pre-treated with or without MT (0.4 mM) for 5 minutes or 25 µM CQ or 2.5 mM 3MA treatment for 1 hour and then exposed to HT (42 °C for 60 minutes), After HT treatment, MT was washed out, and fresh complete medium was added before incubation at 37 °C for 24 hours. (D) Cells were loaded with GFP-RFP-LC3 for 24 hours and then treated with Protocols, analyzed using fluorescence microscope. Red puncta (RFP puncta only) and yellow puncta (RFP puncta colocalized with GFP puncta). (E) Quantification of puncta with brightness values exceeding 60 was done using a Python script. (F) (G) LC3 proteins was determined using western blotting. (H) Cells were loaded with Lysotracker Red DND-99 for 30 minutes and analyzed using fluorescence microscope. (I) Quantification of lysosome red puncta was done using the FIJI program. (J) LAMP 1 and Cathepsin D proteins was determined using western blotting. (K) Quantification of immunoblotting data was performed using the FIJI program, normalized to β-actin as a loading control. The data in each bar graph are presented as the means standard errors of the means (SEM). *P < 0.05, **P < 0.01, ***P < 0.001.

ER stress and autophagy are tightly interconnected [41]. Autophagy is a cellular recycling process where cells break down and reuse their own components [5]. This involves forming structures called autophagosomes, which engulf and deliver misfolded proteins and damaged organelles to lysosomes for degradation [4]. In our study, we found that MT combined with HT can induce autophagic response in HeLa cells. Specifically, we used immunofluorescence staining to observe the expression of the LC3 protein, a marker of autophagy, in treated cells. We observed a significantly increased in the aggregation of LC3 fluorescent puncta in the group subjected to combined treatment compared with HT treated alone (Fig. 5A). Additionally, western blotting results showed a marked increase in LC3-II protein levels in the combined treatment group, the levels of ATG5, did not show significant alteration (Fig. 5B). These findings suggest that the combination of MT and HT effectively stimulates autophagic responses in HeLa cells.

During the progression of cancer, autophagy assists cancer cells in adapting to the harsh tumor microenvironment, such as hypoxia and nutrient deprivation, thereby promoting cancer development and resistance to therapy. In certain types of cancer, inhibiting autophagy can increase the sensitivity of cancer cells to chemotherapy and radiotherapy [42]. For instance, chloroquine and hydroxychloroquine are commonly used autophagy inhibitors that can block the autophagic process and enhance the efficacy of anticancer treatments [43]. Therefore, we investigated the autophagic flux status. At first, we analyzed the expression of Beclin-1 and p62 proteins using western blotting, the results indicated that combined treatment significantly increased the levels of p62 in HeLa cells and decreased the levels of Beclin-1 (Fig. 5C), indicating that autophagic flux was inhibited. Next, we compared the ratio of LC3-II to β-actin in cells treated with combined treatment to untreated cells, both in the presence and absence of the early-stage autophagy inhibitor 3-MA or the autophagosome-lysosome fusion inhibitor CQ. The results show that coincubation of the combination treatment with 3-MA decreased the LC3-II/β-actin ratio (Fig. 5F–5G). Conversely, coincubation with CQ resulted in an LC3-II/β-actin ratio comparable to that of cells treated with the combination treatment. These findings suggest that the combination treatment may impairs the late stage of autophagic flux. To further explore the role of combination treatment in autophagy, we used the Premo™ Autophagy Tandem Sensor RFP-GFP-LC3B to transfect cells. GFP fluorescence is quenched in acidic environments, while RFP fluorescence remains stable. Upon autophagosome formation, the overlapping green and red emissions produce a yellow signal. As autophagosomes merge with lysosomes, forming autolysosomes with acidic interiors, GFP fluorescence is quenched, while RFP remains luminescent. From the results, it is evident that the combined treatment group and the CQ group exhibited similar performance, with no noticeable reduction in green fluorescence (Fig. 5D–5E). These results suggest that combination treatment inhibits later stages of autophagy.

Moreover, we investigated the potential effect of MT combined with HT on lysosomal functionality. Initially, we measured the effect of MT combined with HT on lysosomal pH in HeLa cells using LysoTracker Red DND-99 fluorescent probe. Results showed that the LysoTracker Red DND-99 fluorescence in cells treated with combination treatment significantly decreased compared with HT treated alone (Fig. 5H–5I). Next, we analyzed the expression of LAMP 1 and Cathepsin D proteins through western blotting. The results indicated that the combined treatment decreased the expression of LAMP 1 and Cathepsin D proteins in HeLa cells compared to HT treatment alone (Fig. 5J–5K), suggesting that the combined treatment impacts lysosomal function. These results suggest that combination treatment affects lysosomal function and increase the pH value of lysosomes.

### MT combined with HT induced autophagy counter apoptosis

**Fig. 6:**
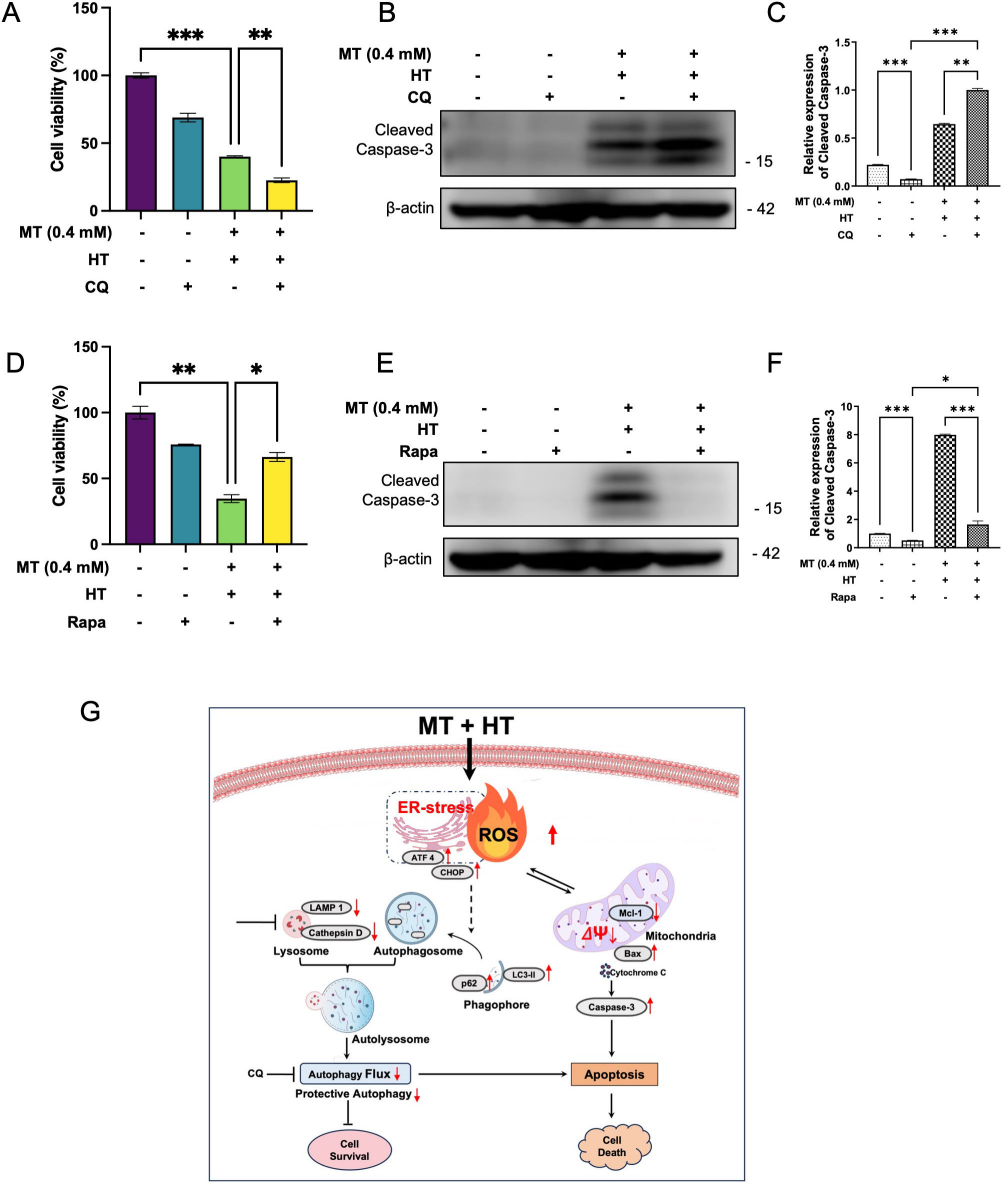
MT combined with HT induced autophagy counteracting apoptosis. Cells were pre-treated with 25 µM CQ, an autophagy inhibitor or 50 µM Rapa, an autophagy inducer for 1 hour and exposed to HT (42 °C; 60 minutes) in the presence of MT (0.4 mM). (A) (D) Cell viability analysis was performed using Cell Counting Kit-8 assay after 24 hours. (B) (E) Cleaved Caspase-3 protein was determined using western blotting. (C) (F) Quantification of immunoblotting data was performed using the FIJI program, normalized to β-actin as a loading control. (G) Mechanism diagram of MT combined with HT induce HeLa cell death. The data in each bar graph are presented as the means standard errors of the means (SEM). *P < 0.05, **P < 0.01, ***P < 0.001.

To further examine the role of autophagy in enhancing apoptosis induced by MT and HT, we utilized CQ, an autophagy inhibitor, and Rapa, an autophagy activator, to explore the correlation between autophagy and MT combined with HT-induced apoptosis in HeLa cells. Subsequent analysis revealed that the autophagy inhibitor CQ reduced HeLa cells viability following the combined treatment (Fig. 6A), while the autophagy activator Rapa improved cell viability under the same conditions (Fig. 6D). Next, we evaluated the expression of Cleaved Caspase-3 protein in the combined treatment group with the addition of autophagy inhibitors or activators using western blotting. The results showed that the introduction of CQ upregulated Cleaved Caspase-3 expression (Fig. 6B–6C), whereas Rapa downregulation Cleaved Caspase-3 expression (Fig. 6E–6F). These findings suggest that impaired autophagy contributes to enhanced apoptosis.

### MT combined with HT showing relatively lower toxicity in HEK293 cells

To further evaluate the potential clinical relevance of combining MT with HT, we examined its cytotoxic and apoptotic effects in a normal human epithelial cell line (HEK293). HEK293 cells were treated with or without MT (0.4 mM) and/or hyperthermia (42 °C) for 1 hour. After HT treatment, MT was washed out, and the cells were incubated in fresh medium for an additional 24 hours. Cell viability was assessed using the CCK-8 assay, while apoptosis was evaluated by Annexin V-FITC/PI double staining followed by flow cytometry. Mitochondrial membrane potential (MMP) was analyzed using the TMRM fluorescent probe, also detected by flow cytometry. Additionally, the expression levels of apoptosis-related proteins, including Caspase-3, Cleaved Caspase-3, Bid, and MCL-1 were examined by Western blotting. These results demonstrate that MT combined with HT significantly less toxic to HEK293 cells than HeLa cells (Supplementary Fig. S1A–S1D). These results strengthen claims of cancer-selective cytotoxicity and support the therapeutic window.

## Discussion

Cancer treatment has always been a topic of great concern, and hyperthermia therapy has recently gained significant attention as a potential method for tumor treatment. This therapy works by raising the local temperature (typically within the range of 41–45 °C), inducing apoptosis of tumor cells or enhancing the effects of other treatment modalities [7, 44, 45]. However, hyperthermia therapy alone faces several limitations, including damage to normal tissues and induction of hyperthermia resistance in cancer cells [46]. Therefore, to find sensitizers that enhance the effectiveness of hyperthermia therapy is of great clinical significance [21, 47]. In this study, we demonstrate that the combination of MT and hyperthermia significantly enhances apoptosis in HeLa cells. The mechanism is associated with the induction of excessive oxidative stress, ER stress, mitochondrial dysfunction, lysosomal dysfunction and impairment of autophagy flux.

Our study shows that the combination of MT and HT significantly suppresses proliferation and induce apoptosis in HeLa cells. We found that, after combined treatment with MT and HT, compared to the groups treated individually, the expression of MCL-1 protein significantly decreased, while the expression of Bax protein significantly increased and full-length Bid significantly decreased. There was no significant change in Bcl-2 protein expression. Additionally, an increase in intracellular ROS levels and mitochondrial dysfunction was confirmed by a decrease in mitochondrial membrane potential [34, 48]. Therefore, the combination of MT and HT could induce mitochondrial dysfunction, thereby inducing mitochondrial-dependent apoptotic pathways. Notably, total Sirt-3 and SOD-2 protein levels did not decline further in the MT + HT group compared with the HT-alone group (Fig. 3E, 3F). Therefore, the additional ROS burst and mitochondrial dysfunction observed after the combined treatment are unlikely to be driven by an extra down-regulation of these antioxidant proteins. Instead, post-translational events—such as altered Sirt-3 de-acetylase activity or changes in SOD-2 acetylation/activation—may underlie the early oxidative stress. Future studies will measure enzyme activity, acetylation status, and longer time-course changes in total protein to test this possibility. Mitochondria are crucial sites for cellular metabolism and energy production. They play a pivotal role in the apoptotic pathways [34]. Changes in mitochondrial membrane permeability lead to the release of substances such as cytochrome c, which, upon release, forms apoptotic bodies with other proteins, activates caspase enzymes, and induces cell apoptosis [49]. Anti-apoptotic proteins Bcl-2 and MCL-1 can inhibit changes in mitochondrial membrane permeability, thereby inhibiting cell apoptosis. On the other hand, pro-apoptotic proteins Bax and Truncated Bid (t-Bid) induce changes in mitochondrial membrane permeability, leading to cell apoptosis [35, 50–52]. Oxidative stress and mitochondrial dysfunction play a crucial regulatory role in processes of the apoptosis induced by MT and HT.

Interestingly, we also observed that the combination of MT and HT induced ER stress, as evidenced by increased expression of p-PERK/PERK, CHOP and ATF-4 proteins. The PERK-ATF4-CHOP pathway is a crucial cellular stress response pathway primarily activated under conditions of ER stress. CHOP is a key regulatory factor in the ER stress response. When the ER stress signaling pathway is activated, CHOP expression is significantly upregulated [53]. It participates in regulating the cell’s response to stress by modulating the expression of relevant genes, such as promoting the expression of apoptotic genes and inhibiting cell growth and proliferation. ATF-4 is another key regulatory factor in the ER stress response. ATF-4 primarily regulates a series of genes involved in amino acid metabolism, protein synthesis, antioxidant responses, and more to meet the cell’s demands in responding to stress, and also upregulate CHOP express [54]. Additionally, in our study, we found that the combination of MT and HT increased the expression of p-JNK and p-p38 proteins in cells. p-JNK and p-p38 are members of the MAPK family and play important roles in mediating ER stress and apoptosis processes [12, 40]. MT therefore enhances cellular sensitivity to apoptosis and improve the therapeutic efficacy of HT by inducing ER stress and activating the MAPK pathway.

Furthermore, our study shows that the combination of MT and HT activated autophagy, evidenced by increases in the expression of LC3-II proteins. Autophagy is intricately linked to ER stress, with sustained or unresolved ER stress often triggering autophagy as a mechanism to restore cellular homeostasis. Under ER stress dysregulation of autophagy can lead to apoptotic cell death [4, 42]. The p62 protein serves as an autophagy adapter protein, linking organelles or proteins targeted for degradation to autophagosomes [55]. An increase in intracellular p62 levels may indicate inhibited or dysfunctional autophagic flux, resulting in decreased autophagic activity [56]. Our results show that and an increase in p62 protein expression, indicating that the autophagic flux is inhibited, which suggests a complex role for the combination of MT and HT in regulating the cellular autophagic process. Moreover, the Autophagy Tandem Sensor RFP-GFP-LC3B system results demonstrated autophagosomes were not degraded, further confirm impaired autophagic flux, suggesting that the combined treatment of MT and HT impair lysosomal function or the autophagic degradation pathway.

PERK activates the ER stress, promoting autophagy in lung cancer cells after heat stress. However, inhibiting autophagy can enhance the apoptosis of lung cancer cells induced by high temperature (HT) [57]. Another study indicated that the autophagy inhibition effect of chloroquine significantly inhibits the growth of hepatocellular carcinoma cells and enhances the apoptotic effect induced by in vivo heat treatment [58]. In our study, increased ER stress and impaired autophagic flux were induced by the combined treatment of MT and HT. Additionally, CQ further reduced HeLa cell viability and upregulated Cleaved Caspase-3 expression following the combined treatment, whereas Rapa improved cell viability and downregulated Cleaved Caspase-3 expression under the same conditions. These results suggest that increased ER stress and autophagic flux blockage contribute to enhanced apoptosis.

Moreover, the expression levels of LAMP 1 and cathepsin D proteins were significantly reduced in the combination of MT and HT group compared to the HT alone group. Autophagosomes and lysosomes can merge under certain conditions to form autolysosomes, where the substances engulfed by autophagosomes are degraded by lysosomal enzymes, completing the thorough degradation and clearance of materials [59]. LAMP 1 is an important lysosomal membrane protein that plays a crucial role in maintaining lysosomal function and promoting the fusion of autophagosomes with lysosomes [60]. Lysosomes have an acidic environment, typically with a pH around 4.5 to 5.0, under the acidic environment cathepsin D contributes to the breakdown of autophagic substrates within autolysosomes [61, 62]. Deficiency or dysfunction of cathepsin D can lead to the accumulation of undigested substrates, thereby impairing autophagic flux and contributing to cellular stress and pathology [63].

To further evaluate the potential clinical relevance of this combination strategy, we investigated its effects on a normal human epithelial cell line (HEK293). Our results revealed that MT combined with HT induces markedly lower cytotoxicity and apoptosis in non-cancerous HEK293 cells compared to HeLa cancer cell line (Fig. S1A–S1D). This selective tolerance compared with HeLa cancer cells, underpins a favorable therapeutic window. However, further in vivo studies will be necessary to confirm these findings and assess treatment safety in a physiological context. Additionally, mechanistic investigations, such as gene knockdown or overexpression of key regulatory proteins, will be important to elucidate the precise molecular pathways responsible for the observed synergy between MT and HT.

## Conclusion

Our study revealed multiple mechanisms through which MT acts as a sensitizing agent for HT. MT increased ER stress, induced mitochondrial dysfunction, elevated ROS levels, and disrupted autophagic flux, thereby synergistically enhancing HT-induced apoptosis. These findings provide crucial clues for further understanding the mechanism of action of MT in hyperthermia treatment and offer scientific evidence for its potential clinical application. However, despite the significant results achieved in vitro, further in vivo and clinical research is needed to validate these mechanisms and determine the optimal strategy for combining MT and HT.

## Abbreviations

4-PBA: 4-phenylbutyric acid
ATF4: Activating transcription factor 4
ATG5: Autophagy-related gene 5
Bak: Bcl-2 homologous antagonist killer
Bax: Bcl-2-associated X protein
Bcl-2: B-cell lymphoma 2
Bid: BH3-interacting domain death agonist
Caspase-3: Cysteine-aspartic protease 3
CCK-8: Cell Counting Kit-8
CHOP: C/EBP homologous protein
CQ: Chloroquine
DAPI: 6-diamidino-2-phenyl-indole dihydrochloride
DCFH-DA: 2′,7′-Dichlorodihydrofluorescein diacetate
DHE: Dihydroethidium
ER: Endoplasmic reticulum
FITC: Fluorescein isothiocyanate
HSPs: Heat shock protein
IRE1: Inositol-requiring enzyme 1
JNK: c-Jun N-terminal kinase
LAMP 1: Lysosomal-associated membrane protein 1
LC3-II: Microtubule-associated protein 1A/1B-light chain 3-II
MAPKs: Mitogen-activated protein kinases
MCL-1: Myeloid cell leukemia 1
Mito-TEMPO: [2-[(1-Hydroxy-2,2,6,6-tetramethylpiperidin-4-yl) amino]-2-oxoethyl]-triphenylphosphanium
MMP: Mitochondrial membrane potential
MOMP: Mitochondrial outer membrane permeabilization
NAC: N-acetyl-L-cysteine
PERK: Protein kinase R-like endoplasmic reticulum kinase
PI: Propidium iodide
p-JNK: Phosphorylated c-Jun N-terminal kinase
p-PERK: Phosphorylated protein kinase R-like endoplasmic reticulum kinase
p62: Sequestosome 1
PVDF: Polyvinylidene fluoride
Rapa: Rapamycin
ROS: Reactive oxygen species
TMRM: Tetramethylrhodamine methyl ester
UPR: Unfolded protein response
Z-VAD-FMK: Z-Val-Ala-Asp(OMe)-fluoromethylketone

## Supplementary information

**Additional file 1:** **Table S1.** Cell viability (mean ± SD, n = 3) under different concentrations of MT. **Table S2.** Cell viability (mean ± SD, n = 3) under different treatment conditions. **Supplementary Fig. S1** HEK293 cells were pre-treated with or without MT (0.4 mM) for 5 minutes and then exposed to HT (42 °C for 60 minutes) or not, then MT washed out after HT treatment and incubated in fresh medium at 37 °C. After 24 hours, cell viability analysis was performed using Cell Counting Kit-8 assay (A), apoptosis detected by flowcytometry using Annexin V-FITC/PI Apoptosis Detection Kit (B), MMP detected by flowcytometry using TMRM fluorescent probe (C), the expression levels of total Caspase-3, Cleaved Caspase-3, Bid and MCL-1 proteins was determined using western blotting (D). The data in each bar graph are presented as the means standard errors of the means (SEM). *P < 0.05.

## Declarations

### Availability of data and materials

All data are incorporated into the article.
