# Supplementary material for: Synergistic inhibition of autophagic flux and induction of apoptosis in cervical cancer cells by Mito-TEMPO and hyperthermia
Source: Environ Health Prev Med. 2025 Sep 5;30:67. doi: 10.1265/ehpm.25-00204 (PMC12436067; doi:10.1265/ehpm.25-00204)
Supplement: Supplementary file 1 — Additional file 1: Table S1. Cell viability (mean ± SD, n = 3) under different concentrations of MT. Table S2. Cell viability (mean ± SD, n = 3) under different treatment conditions. Supplementary Fig. S1 HEK293 cells were pre-treated with or without MT (0.4 mM) for 5 minutes and then exposed to HT (42 °C for 60 minutes) or not, then MT washed out after HT treatment and incubated in fresh medium at 37 °C. After 24 hours, cell viability analysis was performed using Cell Counting Kit-8 assay (A), apoptosis detected by flowcytometry using Annexin V-FITC/PI Apoptosis Detection Kit (B), MMP detected by flowcytometry using TMRM fluorescent probe (C), the expression levels of total Caspase-3, Cleaved Caspase-3, Bid and MCL-1 proteins was determined using western blotting (D). The data in each bar graph are presented as the means standard errors of the means (SEM). *P < 0.05. [file ehpm-30-067-s001.docx]

**Supplementary Table**

**Table S1.** Cell viability (mean ± SD, n = 3) under different concentrations of MT.

| **Concentration of MT** | **Cell viability (Mean ± SD %)** |
| --- | --- |
| 0 mM | 100.00 ± 3.33 |
| 0.05 mM | 95.83 ± 3.55 |
| 0.1 mM | 99.49 ± 3.23 |
| 0.25 mM | 86.66 ± 5.96 |
| 0.5 mM | 39.82 ± 7.47 |
| 1 mM | 1.05 ± 0.70 |

HeLa cells were continuously exposed to various concentrations of MT for 24 hours. Cell viability analysis was performed using Cell Counting Kit-8 assay.

**Table S2.** Cell viability (mean ± SD, n = 3) under different treatment conditions.

| **Group** | **Cell viability (Mean ± SD %)** |
| --- | --- |
| Control | 100.00 ± 1.00 |
| MT (0.4 mM) | 86.13 ± 4.00 |
| HT | 80.25 ± 2.03 |
| MT (0.4 mM) + HT | 42.76 ± 7.56 |

Cells were pre-treated with or without MT (0.4 mM) treatment for 5 minutes and then exposed to HT (42°C for 60 minutes) or not, then wash out MT after HT treatment and incubated in fresh medium at 37˚C. Cell viability analysis was performed using Cell Counting Kit-8 assay after 24 hours.

**Supplementary Figure**

**
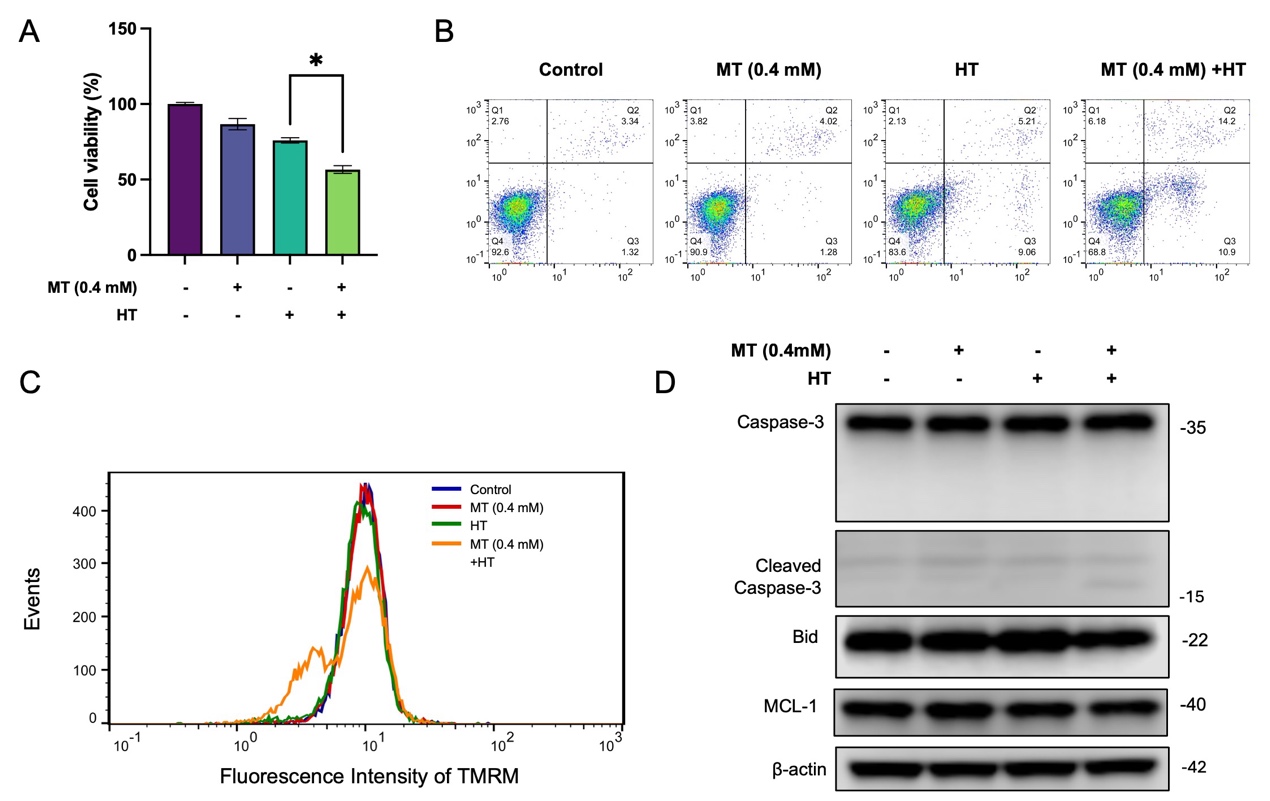
**

**Supplementary Fig. S1** HEK293 cells were pre-treated with or without MT (0.4 mM) for 5 minutes and then exposed to HT (42°C for 60 minutes) or not, then MT washed out after HT treatment and incubated in fresh medium at 37˚C. After 24 hours, cell viability analysis was performed using Cell Counting Kit-8 assay (A), apoptosis detected by flowcytometry using Annexin V-FITC/PI Apoptosis Detection Kit (B), MMP detected by flowcytometry using TMRM fluorescent probe (C), the expression levels of total Caspase-3, Cleaved Caspase-3, Bid and MCL-1 proteins was determined using western blotting (D). The data in each bar graph are presented as the means standard errors of the means (SEM). *P<0.05.
